# Antibiotic tolerance and persistence have distinct fitness trade-offs

**DOI:** 10.1371/journal.ppat.1010963

**Published:** 2022-11-14

**Authors:** Charlotte Michaux, Séverin Ronneau, Rachel T. Giorgio, Sophie Helaine

**Affiliations:** Department of Microbiology, Harvard Medical School, Boston, Massachusetts, United States of America; Stanford University School of Medicine, UNITED STATES

## Abstract

Genetically susceptible bacteria can escape the action of bactericidal antibiotics through antibiotic tolerance or persistence. However, one major difference between the two phenomena is their distinct penetrance within an isogenic population. While with antibiotic persistence, susceptible and persister cells co-exist, antibiotic tolerance affects the entire bacterial population. Here, we show that antibiotic tolerance can be achieved in numerous non-specific ways *in vitro* and during infection. More importantly, we highlight that, due to their impact on the entire bacterial population, these tolerance-inducing conditions completely mask persistence and the action of its molecular determinants. Finally, we show that even though tolerant populations display a high survival rate under bactericidal drug treatment, this feature comes at the cost of having impaired proliferation during infection. In contrast, persistence is a risk-limiting strategy that allows bacteria to survive antibiotic treatment without reducing the ability of the population to colonize their host. Altogether, our data emphasise that the distinction between these phenomena is of utmost importance to improve the design of more efficient antibiotic therapies.

## Author summary

Even if antibiotics should eradicate entirely a genetically susceptible bacterial population, some cells escape the action of the drugs. This phenomenon is assumed to cause antibiotic treatment failure and infection relapse. Antibiotic recalcitrance is known to operate either on the entire population (antibiotic tolerance) or on a subset of cells (antibiotic persistence). Here, we show how conditions that induce bacterial tolerance can mask entirely persistence and its molecular determinants in the pathogen *Salmonella* Typhimurium. The different fitness trade-offs associated with antibiotic tolerance and persistence have important consequences during infection. Understanding the distinct dynamics of these modes of recalcitrance will allow the design of more efficient antibiotic approaches.

## Introduction

Antibiotic treatment failure can be attributed to the presence of resistant bacteria that proliferate and spread in the presence of a specific antibiotic, or of genetically-susceptible bacteria that transiently escape the action of antibiotics. The latter phenomenon, referred to as antibiotic recalcitrance or resilience, has been observed in many bacterial species, including various pathogens such as *Mycobacterium tuberculosis* [1], *Staphylococcus aureus* [2], *Yersinia pseudotuberculosis* [3] and *Salmonella enterica* [4,5]. These recalcitrant cells are slow—or non-growing bacteria, which may cause infection relapse following withdrawal of the antibiotic [6]. Importantly, these bacteria also participate in the emergence [7,8] and spread [9] of antibiotic resistance.

Antibiotic recalcitrance can be displayed by a subpopulation of cells within a fully susceptible population (antibiotic persistence) or the entire population (antibiotic tolerance) [10]. At the single bacterial cell level, both phenomena seem similar with growth-restricted cells escaping the action of antibiotics. As such, studying antibiotic tolerance has been sometimes used as a convenient substitute to investigate the molecular mechanisms underlying antibiotic persistence. However, recent advances in our understanding of antibiotic persistence shed some light on the distinct nature of these two modes of survival [11].

Notably, persisters are characterized by their co-existence with growing counterparts in environments that are thus, by extension, permissive for growth. Hence, they are often considered as a pool of cells set aside that maximise the chances of survival of the population in case of changes in the environment. By contrast, antibiotic tolerance is achieved under environmental or genetic conditions that are uniformly restrictive for growth, resulting in antibiotics being inefficient against the entire population. Accordingly, molecular determinants involved in antibiotic persistence may be dispensable in tolerance-inducing conditions. For instance, RecA, as a main actor of the double strand DNA break repair machinery, is required for persister survival in *Salmonella* during infection [12] but is dispensable in a highly tolerant strain [12,13]. This example highlights the importance of understanding the differences between antibiotic persistence and tolerance during infection to adapt future therapeutic strategies accordingly.

Here, by quantifying antibiotic survival and by monitoring the growth status of the population at the single cell level, we illustrate how mutations and environmental conditions that induce *Salmonella* tolerance may mask antibiotic persistence both *in vitro* and in the context of infection. In addition, we show that due to their differential penetrance in the population, antibiotic persistence and tolerance have distinct fitness trade-offs. Our data show that antibiotic persistence is a more balanced strategy than tolerance, providing an equilibrium that allows survival and colonization both in the presence and absence of antibiotic treatment.

## Results

### Antibiotic tolerance masks persistence in non-permissive growth conditions

**Fig 1:**
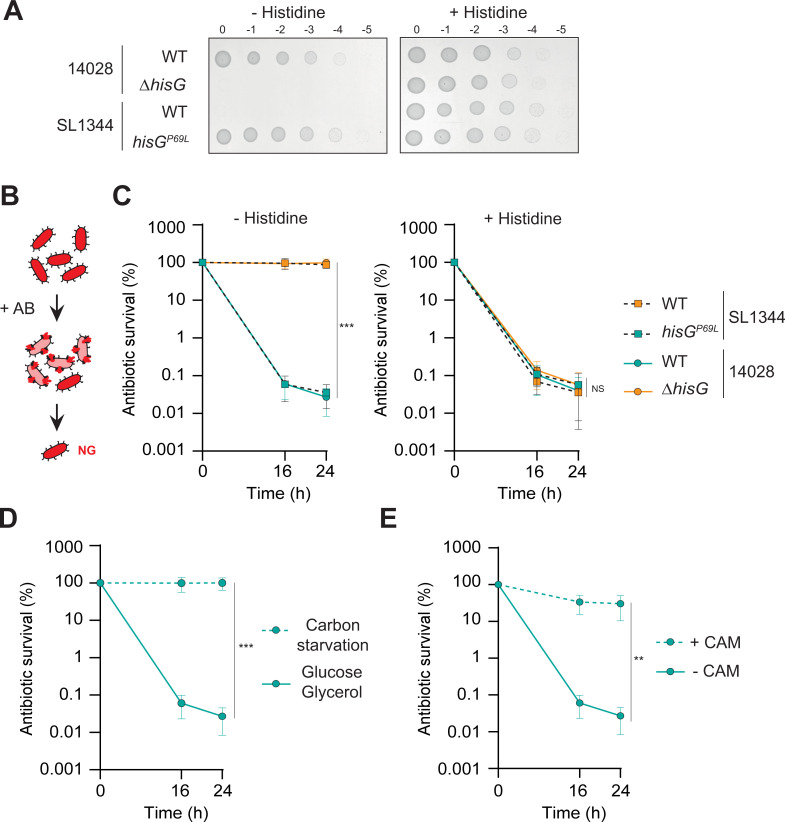
Genetic and/or environmental factors can lead to antibiotic tolerance in laboratory medium. (**A**) Growth of WT and *ΔhisG* 14028 and WT and *hisG*^P69L^ SL1344 *Salmonella*. Ten-fold dilution series were spotted on minimal medium plates in the absence (left) or presence (right) of histidine. (**B**) Illustration of the *in vitro* antibiotic survival assay where growers (pink) are killed by cefotaxime and non-growers (red) survive. (**C**) CFU enumeration of survival of WT and *hisG*^P69L^ SL1344 *Salmonella* as well as WT and *ΔhisG* 14028 *Salmonella* exposed to cefotaxime treatment in M9GG in absence (left) or presence (right) of histidine. Data from the 24 h timepoint were compared using one way ANOVA with Tukey’s multiple-comparison test, ***p<0.001; NS, not significant. (**D**) CFU enumeration of survival of WT 14028 exposed to cefotaxime in minimal medium supplemented with carbon sources (Glucose/Glycerol) or not (carbon starvation). (**E**) CFU enumeration of survival of WT 14028 exposed to cefotaxime in M9GG medium in the presence or absence of chloramphenicol (CAM). (**D-E**) Statistical significant differences by two-sided *t*-test between the 24 h timepoint are indicated as **p<0.01; NS, not significant. Panels **C-E**, data represent the mean and standard deviation (SD) of at least three biological repeats. Of note, WT data are the same on panel **D** and **E** as all experiments were conducted in parallel. Auxotrophic strains are depicted in orange, prototrophic in turquoise.

Most published studies on *Salmonella enterica* serovar Typhimurium (henceforth *Salmonella*) have focused on two strains, SL1344 and ATCC 14028 (also known as NCTC 12023). It is commonly assumed that data obtained with one are representative of both Typhimurium strains. However, some important differences exist between the two strains. For instance, in contrast to 14028, histidine biosynthesis is inactive in the SL1344 strain because of a non-functional *hisG*^Pro69^ allele [14,15]. Accordingly, whereas both 14028 and SL1344 grew well on agar plates containing histidine, we observed no growth of wild-type (WT) SL1344 in the absence of histidine. The *hisG* deletion mutant of 14028 behaved like WT SL1344, whereas restoration of the functional allele (*hisG*^P69L^) in SL1344, rescued growth in absence of histidine [16] (Fig 1A). We therefore predicted that antibiotic susceptibility of the two WT strains would diverge in histidine poor environments, such as those encountered in the host during infection [14,17].

To determine the impact of histidine on antibiotic survival of WT 14028 and SL1344 *Salmonella* strains, we challenged them both with cefotaxime, a β-lactam antibiotic targeting actively growing cells (Fig 1B), in the presence or absence of histidine in minimal laboratory medium. As expected based on its inability to grow in the absence of histidine, most of the WT SL1344 population survived cefotaxime over 24 h in histidine deprived medium (Fig 1C). In contrast, the WT 14028 strain exhibited biphasic killing kinetics where the first slope represented rapid killing of most of the population, and the second slope revealed a small subpopulation of persister cells killed much more slowly. The restored prototrophic *hisG*^P69L^ mutant in SL1344 was re-sensitized to antibiotic, displaying a killing profile similar to that of the WT 14028 strain. Conversely, deletion of *hisG* in the 14028 strain resulted in the entire population surviving bactericidal treatment similarly to WT SL1344. All strains displayed biphasic killing in medium containing histidine (Fig 1C). These results show that the histidine auxotrophy of SL1344 causes antibiotic tolerance *in vitro* in the absence of histidine, thus masking the presence of any persister subpopulation.

Histidine auxotrophy is not a unique path to antibiotic tolerance, as any bacteriostatic condition favours bacterial survival to β-lactam antibiotics [18–21]. Accordingly, WT 14028 *Salmonella* challenged with cefotaxime in two bacteriostatic conditions (carbon starvation or in the presence of chloramphenicol), displayed antibiotic tolerance, similar to what was observed with the auxotrophic strains (Fig 1D and 1E). Thus, just as the histidine auxotrophy of SL1344 in a histidine-deprived environment masks the persister subpopulation, so too do other bacteriostatic conditions.

**Fig 2:**
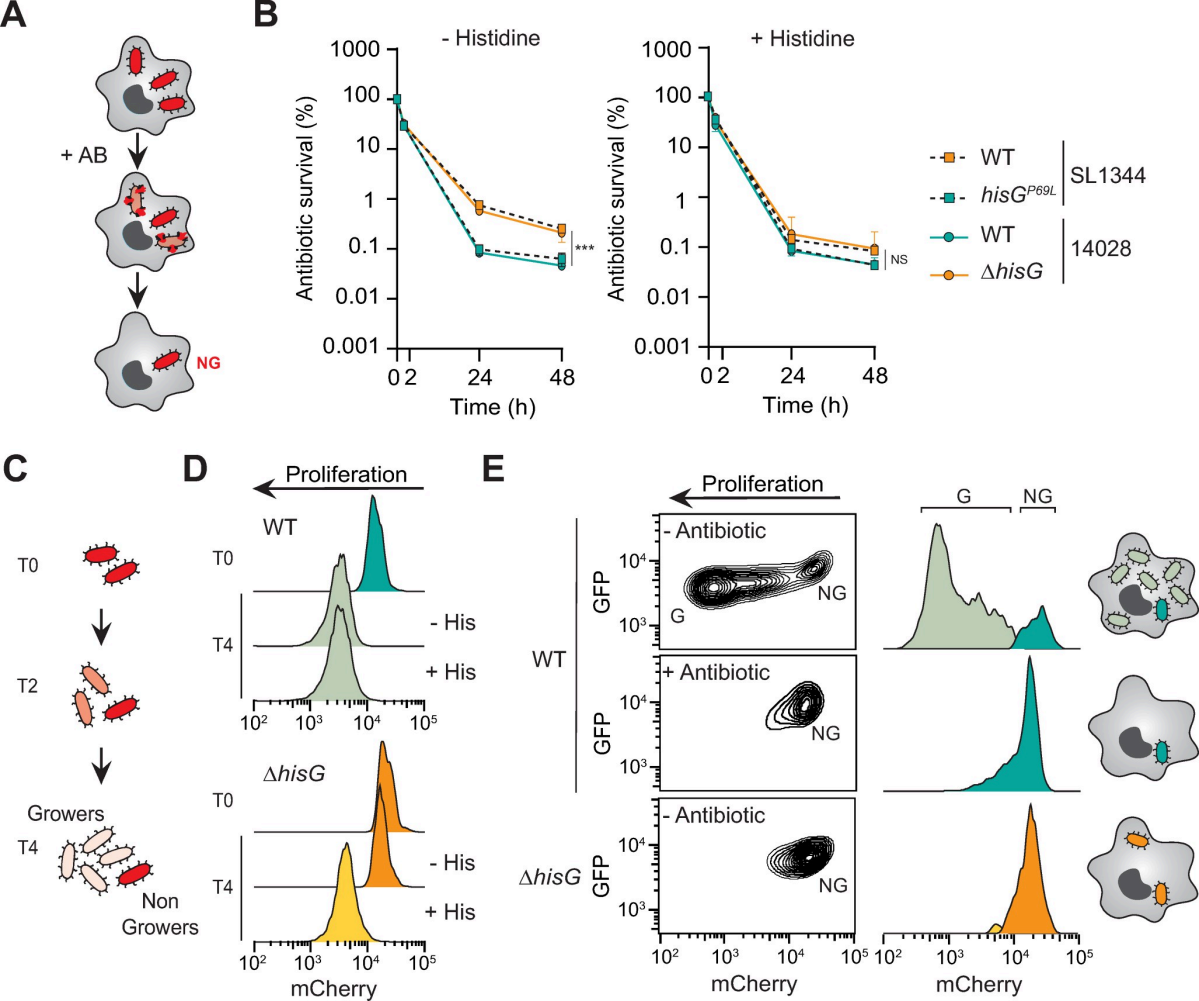
Growth dynamics at the single cell level discriminates antibiotic tolerance from persistence *in cellulo*. (**A**) Illustration of the *in cellulo* antibiotic survival assay in primary Bone Marrow Derived Macrophages (BMDM) infected with *Salmonella*. (**B**) CFU enumeration of WT or *hisG*^P69L^ SL1344 *Salmonella* as well as WT or *ΔhisG* 14028 *Salmonella* in BMDMs treated with cefotaxime in the absence or presence of histidine in the infection medium. Data represent the mean and SD of at least three biological repeats. Data from the 48 h timepoint were compared using one way ANOVA with Tukey’s multiple-comparison test, ***p<0.001; NS, not significant. (**C**) Illustration of the fluorescence dilution assay to assess growth dynamics of a population at the single cell level. Growing cells dilute a pre-formed pool of mCherry at each cell division whereas the non-growing bacteria maintain high intensity fluorescence. (**D**) Representative flow cytometry profile of *Salmonella* 14028 grown in minimal medium for 4 h in the absence or presence of histidine for WT and *ΔhisG* strains. (**E**) Representative flow cytometry contour (left) and histogram (right) plots of bacteria extracted from BMDM after 16 h of gentamicin (- antibiotic condition) or 16 h of cefotaxime (+ antibiotic condition). G, growers and NG, non-growers.

Engulfment of *Salmonella* by macrophages increases the proportion of non-growing antibiotic persistent bacteria present in the population compared to cells grown in laboratory medium [5]. To investigate whether the histidine auxotrophy of SL1344 may also mask the persister population during macrophage infection, we infected murine bone-marrow derived macrophages with the 14028 or SL1344 strains. Infected macrophages were treated with cefotaxime, which readily reaches the intracellular bacterial population [5]. As occurs in laboratory medium, bacteria that proliferate within their host cells are lysed by the antibiotic, whereas the non-growing fraction survives and constitutes the persister subpopulation (Fig 2A). In this assay, strains unable to synthesise histidine (SL1344 and 14028 *ΔhisG*) exhibited higher survival than their prototrophic counterparts (SL1344 *hisG*^P69L^ and 14028) (Fig 2B), indicating that histidine is limiting in host cells, as previously reported [14,17,22]. Remarkably, auxotrophic strains displayed a biphasic-like killing curve, usually thought to reveal antibiotic persistence [10]. To resolve this ambiguity and determine which phenomenon, antibiotic tolerance or persistence, supports recalcitrance of the intracellular population of the auxotrophic strains, we monitored growth dynamics of our different strains at the single cell level using fluorescence dilution (FD) [23]. With FD, all bacteria are pre-loaded with a fluorescent protein whose expression is switched off at the onset of the experiment. Growing bacteria then dilute the pre-existing pool of fluorescent protein, whereas non-growers retain bright fluorescence over time (Figs 2C and S1A). Resolving bacterial growth at the single cell level allows us to determine the heterogeneity of growth in a given population and quantify the proportion of the population that is growth-arrested. Thus, in minimal medium in the presence of histidine the WT and *hisG* deletion mutant diluted the fluorescent signal revealing uniform growth. Of note, the non-growing population in these *in vitro* conditions is too small to be visualized by FD. As expected, absence of histidine in minimal laboratory medium led all bacteria in the auxotrophic *hisG* deletion mutant population to adopt a non-growing state, contrary to the WT which grew uniformly (Fig 2D). As previously described, in macrophages the 14028 WT population of *Salmonella* contained both growing and non-growing bacteria, the latter containing the persisters that escape action of antibiotics [5] (Figs 2E and S1B). In contrast to the growth dynamic heterogeneity observed for the WT, the histidine auxotroph mutant was homogeneously composed of non-growing bacteria, demonstrating that the intracellular population displays antibiotic tolerance rather than persistence (Fig 2E). Expectedly, the auxotrophic SL3144 strain had a similar phenotype than the *hisG* mutant of 14028 and supplementation with exogenous histidine was required to reveal the persister population (S1C and S1D Fig). These results demonstrate that the distinction between antibiotic tolerance and persistence can be greatly facilitated by single cell level measurements of the growth dynamics of the bacterial population in the absence of antibiotics.

What explains the bi-phasic killing observed for the histidine auxotroph mutant in macrophages? First, macrophages participate in the killing of *Salmonella*, especially during the first hours of infection [23], and therefore affect antibiotic survival dynamics. In addition, presence of a limited amount of histidine in the environment could also contribute to our observations. Similar to what we observed *in vitro*, addition of exogenous histidine during the antibiotic treatment decreased antibiotic survival in both auxotrophic strains (Fig 2B). Importantly, addition of histidine did not affect antibiotic survival of the two prototrophic strains (14028 or SL1344 *hisG*^P69L^), demonstrating that histidine limitation does not contribute to the recalcitrance of these strains (Fig 2B). It is also apparent that histidine auxotrophy is the sole genetic determinant of the differential antibiotic recalcitrance of 14028 and SL1344 during macrophage infection.

Taken together, our data show that antibiotic survival of the two workhorse *Salmonella* laboratory strains is indistinguishable in histidine replete conditions. However, they display a different type of antibiotic recalcitrance in histidine poor environments, such as those encountered during infection, with SL1344 being tolerant and 14028 surviving through persistence. Hence, the size of the persister population of SL1344 cannot be measured in histidine limiting conditions.

### Tolerance allows high antibiotic survival that outweighs the effect of genetic persister determinants

**Fig 3:**
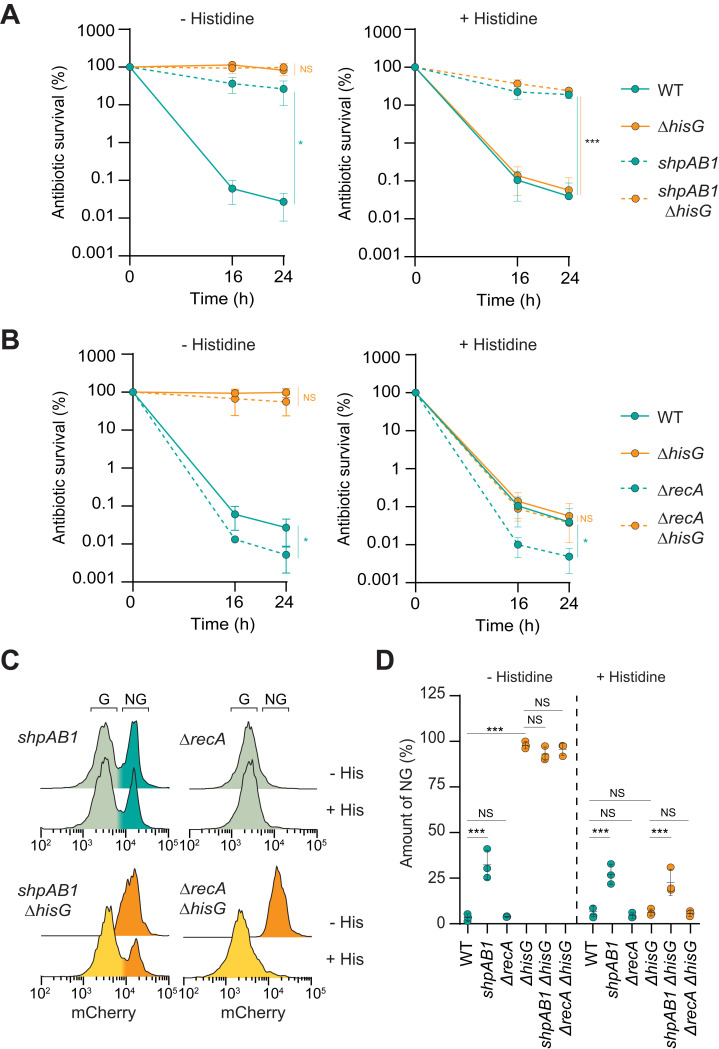
Tolerance can mask persister determinants in laboratory medium. (**A-B**) Survival of *Salmonella* 14028 exposed to cefotaxime in M9GG in the absence or presence of histidine for WT, *ΔhisG*, *shpAB1* and *shpAB1ΔhisG* or WT, *ΔhisG*, *ΔrecA* and *ΔrecAΔhisG*. (**C-D**) Representative flow cytometry profile (**C**) and quantification of the non-grower fraction (D) of *Salmonella* 14028 grown in minimal medium in presence or absence of histidine for *shpAB1*, *ΔrecA*, *shpAB1ΔhisG* and *ΔrecAΔhisG*. (**A-D**) Data represent the mean and SD of at least three biological repeats. Data from the 24 h timepoint were compared using one way ANOVA with Tukey’s multiple-comparison test, *p<0.05, ***p<0.001; NS, not significant. Of note, WT and *ΔhisG* data are the same on panel A and B as all experiments were conducted in parallel. Auxotrophic strains are depicted in orange, prototrophic in turquoise.

Tolerance and persistence have different penetrance in the population, resulting in higher antibiotic survival of populations displaying tolerance than those displaying persistence. This raises the possibility that tolerance can mask the effect of persister determinants. To test this, we chose known hyper- (*shpAB1*) and hypo- (*ΔrecA*) persistence mutations to measure their impact on antibiotic survival and bacterial growth dynamics in a tolerance context. Although we determined that histidine auxotrophy is the sole genetic determinant of the differential antibiotic recalcitrance of 14028 and SL1344, other genes vary between the two strains. We therefore chose to compare 14028 and 14028 *hisG* deletion mutant in histidine limiting environments, as immediately comparable examples of a strain surviving antibiotics through persistence or tolerance, respectively. Thus, we compared the impact of *shpAB1* and *recA* mutations in these two genetic backgrounds (Figs 3A, 3B and S2).

The gain-of-function allele *shpAB1* was selected in a screen for *Salmonella* hyper-persistence *in vitro* [24]. This mutation deregulates the ShpAB toxin-antitoxin module, resulting in a higher fraction of cells surviving β-lactam exposure compared to WT [24,25] (Fig 3A) as well as an increased fraction of non-growing bacteria in the population in laboratory medium (Fig 3C and 3D). However, the *shpAB1* allele had no effect on antibiotic survival of the *hisG* deletion strain while it displayed tolerance in histidine-depleted medium (Fig 3A). In parallel, the bimodal growth distribution observed in the *shpAB1* mutant was masked in the tolerant background (*ΔhisG)* where all the bacteria adopted a non-growing state. Addition of histidine, by suppressing the tolerance phenotype of the *hisG* mutants, revealed the impact of *shpAB1* on antibiotic persistence in this background (Fig 3C and 3D). In contrast to the *shpAB1* allele, loss of *recA*, an essential actor of double-stranded break DNA repair, negatively impacts persister survival in *Salmonella* [12] (Fig 3B). Similar to what we observed with the *shpAB1* allele, the loss of *recA* did not impact antibiotic survival of the histidine auxotroph strain in the absence of histidine (Fig 3B). Intriguingly, even if the survival of the double *recA hisG* deletion mutant was strongly reduced in the presence of histidine, the loss of *hisG* still compensated for that of *recA* in antibiotic persistence in the experimental conditions tested here (Fig 3B). As for the WT strain, the non-grower fraction was too small to be visualized in these conditions (Fig 3C and 3D). Altogether these results show that in laboratory medium, antibiotic tolerance supports such a high antibiotic survival that the effects of persister determinants can be masked.

**Fig 4:**
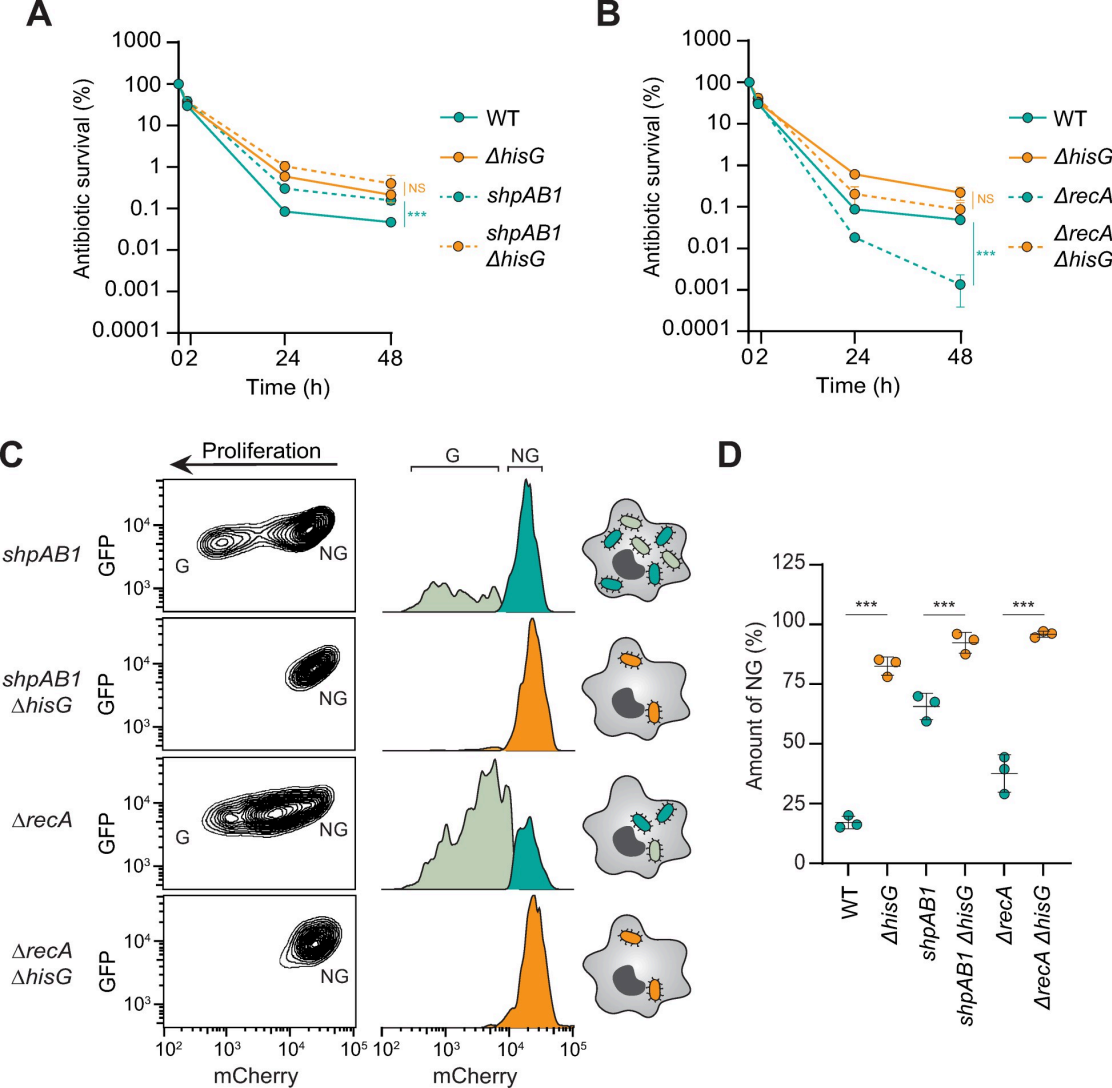
Tolerance can mask persister determinants during macrophage infection. (**A-B**) CFU enumeration of survival of the WT, *ΔhisG*, *shpAB1*, *shpAB1ΔhisG*, *ΔrecA* and *ΔrecAΔhisG Salmonella* strains in BMDMs treated with cefotaxime and in absence of histidine. (**C**) Representative flow cytometry contour (left) and histogram plots (right) of the *shpAB1*, *shpAB1ΔhisG*, *ΔrecA* and *ΔrecAΔhisG* strains extracted from infected BMDM after 16 h of gentamicin. G, growers and NG, non-growers. (**D**) Quantification of the non-grower fraction for each strain represented in panel **C**. Data represent the mean and SD of three biological repeats. Data were compared using one way ANOVA with Tukey’s multiple-comparison test, ***p<0.001; NS, not significant. Of note, WT and *ΔhisG* data are the same on panel A and B as all experiments were conducted in parallel. Auxotrophic strains are depicted in orange, prototrophic in turquoise.

Since infection of host cells imposes more severe constraints on *Salmonella* than laboratory medium conditions, we wondered if antibiotic tolerance could also mask the effects of persister determinants during macrophage infection. In agreement with what we observed in laboratory medium, the *shpAB1* allele resulted in higher intracellular antibiotic survival (Figs 4A and S3A) in line with a higher fraction of non-growing cells in the population compared to the WT (Figs 2E, 4C and 4D). Ectopic expression of *shpB* fully suppressed the hyperpersistence phenotype displayed by the *shpAB1* strain (S3A Fig). In contrast, the impact of the *shpAB1* allele was completely masked in the histidine auxotroph mutant, with *shpAB1ΔhisG* displaying a similar high antibiotic survival and homogeneous non-growing population to *ΔhisG* (Fig 4A, 4C and 4D). Addition of histidine to the infection medium relieved the antibiotic tolerance and growth arrest of the double mutant, revealing the impact of *shpAB1* on antibiotic persistence (S3B Fig). Since growth of the *ΔrecA* mutant is reduced in macrophages (Fig 4C) [12], the relative proportion of intracellular non-growing cells was higher than for the WT strain (Fig 4C and 4D). Nonetheless, the loss of *recA* strongly affects antibiotic persister survival during infection (Fig 4B) [12]. Similar to *in vitro*, the impact of the *recA* mutation on antibiotic survival was completely masked in a tolerant background (Fig 4B). The double mutant completely phenocopied the single *recA* deletion mutant in the presence of histidine, revealing the importance of RecA on antibiotic persistence during infection (S3C Fig). Macrophage infection, these results show that due to its impact on the entire population, antibiotic tolerance can mask the impact of persister determinants in vitro and in the context of macrophage infection.

### Tolerance and persistence have different fitness trade-offs during infection

Our results so far suggest that because of its high penetrance, antibiotic tolerance is a much more effective means of drug survival than antibiotic persistence, which only allows the survival of a few bacteria. However, the flip side of tolerance is that it imposes a restricted growth rate to all cells in the population, which might explain why tolerance mutations were not commonly selected for following cyclical antibiotic exposure of several different bacterial pathogens [12,26–31]. One hypothesis is that the fitness cost of tolerance mutations is too high in the absence of antibiotic treatment to be selected for over time.

**Fig 5:**
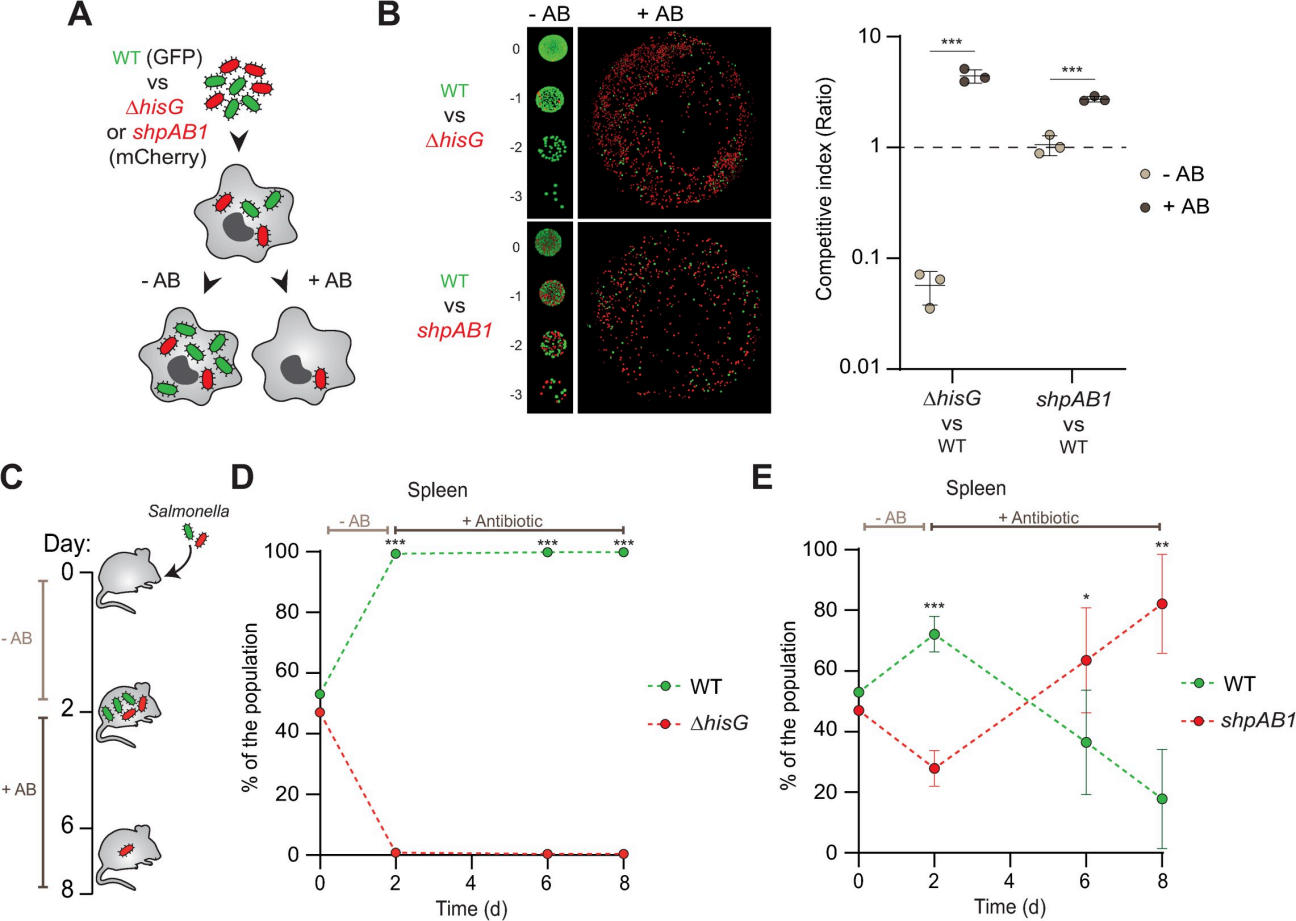
Persistence is a more balanced strategy than tolerance. (**A**) Illustration of the *in cellulo* competition assay where BMDMs were co-infected with the WT/*shpAB1* or WT/*ΔhisG* pair and treated with cefotaxime to assess antibiotic survival (+AB) or gentamicin to assess proliferation (-AB). WT expressed GFP constitutively whereas the *shpAB1* and *ΔhisG* mutants expressed mCherry. (**B**) Left panel, representative images of the bacterial population after extraction of 24 h gentamicin (-AB) or cefotaxime (+AB) treated BMDM. For the proliferation competitive assay (-AB), ten-fold dilution series were spotted on rich medium plate. For the antibiotic survival competitive assay (+AB), the entire bacterial population was plated on rich medium. Right panel, competitive index ratios. Data represent the mean and SD of at least three biological repeats. Statistically significant differences of each pair by two-sided *t*-test are indicated as ***p<0.001. (**C**) Illustration of the *in vivo* mice co-infection assay with the WT/*shpAB1* or WT/*ΔhisG* pair used on panel A and B. Two days after oral gavage, mice were either sacrificed or treated with cefotaxime for 4 or 6 days. (**D-E**) Bacterial survival in the spleen of the WT/ *ΔhisG* (**D**) and WT /*shpAB1* (**E**) mix. Results are expressed in percentage of the total population recovered on plate. Day 0 indicates the inoculum ratio. Statistical significant differences at each timepoint by two-sided *t*-test are indicated as *p<0.05 and ***p<0.001. *In vivo* mice experiments were carried out with at least 4 animals per time point.

To test this hypothesis, we co-infected bone-marrow derived macrophages with the WT strain paired with either a hyper-tolerant (*ΔhisG*) or a hyper-persistent (*shpAB1*) mutant strain to assess their relative fitness in the presence or absence of antibiotic (Fig 5A). In agreement with our observations (Fig 4A and 4B), the *ΔhisG* mutant strongly outcompeted the WT strain in the presence of antibiotic (Fig 5B). However, the absence of growth resulting from histidine auxotrophy (Fig 2E) led the tolerant *ΔhisG* mutant to be outcompeted by the WT strain in macrophages in absence of antibiotic treatment (Fig 5B). This result illustrates the strong fitness cost of antibiotic tolerance during infection. In contrast, the higher antibiotic survival of the *shpAB1* strain in comparison to WT (Figs 4A and 5B) did not affect its competitiveness in absence of antibiotics (Fig 5B).

**Fig 6:**
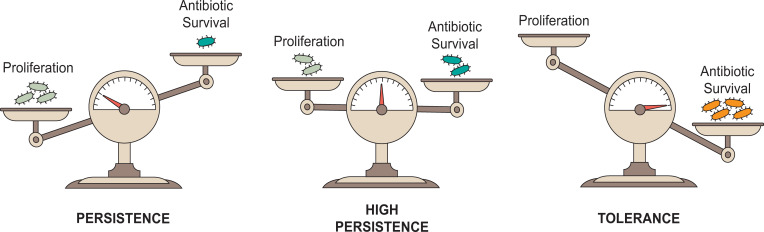
Trade-off between proliferation and survival in antibiotic tolerance and persistence. In the case of antibiotic persistence, the majority of cells grow in the population (light blue), allowing bacteria to efficiently colonize their environment. Persisters (turquoise) represent a small reservoir of recalcitrant cells that outlive their growing counterpart under antibiotic treatment. The frequency of persisters in the population can be increased in some conditions (*e*.*g*. during macrophage infection) or in specific genetic backgrounds (*shpAB1*), at the detriment of the pool of growing bacteria. On the other hand, antibiotic tolerance (orange) strongly reduces the proliferation of the entire population which, in turn, limits the efficacy of the antibiotic treatment on all cells.

To further investigate the effect of the different fitness costs of antibiotic tolerance and persistence in a more complex model, we co-infected C57BL/6 mice orally with WT paired with either the *hisG* deletion or *shpAB1* mutants. After two days of acute infection without antibiotic, we assessed bacterial colonization in the spleen, mesenteric lymph nodes (MLN) and Peyer’s patches (PP) as previously described [12]. To then evaluate the impact of antibiotic treatment on the equilibrium of each bacterial mix, we also treated two other groups of mice with cefotaxime for an additional 4 or 6 days (Fig 5C). As in macrophages, the *ΔhisG* mutant was rapidly outcompeted by the WT strain during the acute phase of the infection (Figs 5D, S4A and S4B). Because of its inability to colonize in the presence of the WT strain, the *ΔhisG* mutant displayed no antibiotic survival advantage in presence of antibiotics (Figs 5D, S4A, S4B and S5A). In contrast to macrophages where the intracellular proliferation of WT and the *shpAB1* strain were comparable, we determined that the WT was more competitive in mice than the *shpAB1* strain in the absence of antibiotic treatment (Figs 5E, S4C, S4D and S5B). However, the lower abundance of the *shpAB1* strain in all tested organs during the acute phase of the infection was not severe enough to prevent bacteria from colonizing all organs and showing a strong survival advantage during the course of antibiotic treatment (Figs 5E, S4C and S4D). These results demonstrate that even if antibiotic tolerance allows dramatic antibiotic survival in certain conditions, the fitness cost associated with its high penetrance in the population can strongly decrease host colonization by the pathogen. On the other hand, populations containing persisters benefit from the ability to both colonize the host and survive antibiotics, emphasizing the advantage of phenotypic heterogeneity when it comes to antibiotic recalcitrance of bacterial infections (Fig 6).

## Discussion

Antibiotic tolerance and persistence are often used interchangeably to describe survival of genetically susceptible cells to bactericidal antibiotics. However, we recently revealed that for the pathogen *Salmonella enterica*, in addition to their differential penetrance in the population, these two phenomena are underpinned by different physiologies [11,12]. Here, we evaluated the interplay and fitness trade-offs of these two strategies *in vitro* and in the context of infection. We found that even if antibiotic tolerance can be considered more advantageous in the face of antibiotic treatment due to its protective effect on the whole population, it comes at the cost of limiting the ability of bacteria to colonize their environment. In contrast, bacterial populations containing persisters, even at higher proportions such as that displayed by the *shpAB1* mutant (Fig 5B), hold the capacity to survive antibiotics while remaining able to establish and proliferate in their niches (Fig 6).

The lack of distinction between antibiotic tolerance and persistence can explain many puzzling contradictions that have appeared in the literature. As a result of their distinct penetrance, we find that conditions leading to drug tolerance hinder the study of antibiotic persistence. For example, transcription or translation inhibitors, such as rifampicin or chloramphenicol have sometimes been used by scientists to maximize the number of antibiotic-recalcitrant cells in a population before further characterization. However, such treatments actually produce tolerant populations that have different phenotypic properties, bypassing the genetic foundations of persistence [19,20]. Likewise, the use of specific genetic backgrounds or mutants can mask the role of key persister determinants. As shown in this work, use of the auxotrophic strain SL1344 can significantly impede the study of antibiotic persistence in histidine-depleted environments such as those encountered by *Salmonella* during infection. This appears especially true in murine models where, although challenging to ascertain the histidine concentration in each niche over time, it is clearly limiting, as demonstrated by the defect that a *hisG* deletion mutant shows compared to a prototrophic counterpart (Fig 5D). As a result, for auxotrophic strains such as SL1344, the source of antibiotic recalcitrance, tolerance or persistence, may vary from organ to organ, where variability in histidine availability in the host may render SL1344 antibiotic tolerance transient or confined to some niches. The distinction matters as several research groups have shown that antibiotic persisters found during infection are non-growing yet active bacteria [5,28,32–35], but this is not necessarily true of tolerant cells [12]. Finally, we demonstrate that even if killing kinetics should be the norm in distinguishing tolerance from persistence in well-controlled experimental conditions [10] (Fig 1), they show some limitations when applied in more complex models. Therefore, pairing killing assays with single cell growth rate reporters such as fluorescence dilution can be extremely useful to determine which phenomenon is the source of antibiotic recalcitrance.

In clinical settings, periodic rounds of antimicrobial therapy have been often associated with the appearance of hyper-persistence [26–31]. Interestingly, a *hipA7* variant, the *E*. *coli* phenotypic equivalent of *Salmonella shpAB1*, was identified in patients with recurrent UTIs [31,36]. However, tolerance mutations have also been reported recently from a patient suffering from Methicillin-resistant *Staphylococcus aureus* (MRSA) bacteraemia and subjected to rounds of antibiotic treatment [37]. These mutations were shown to emerge after colonization of the host, possibly limiting the negative impact tolerance has on bacterial proliferation. Importantly, these mutations were also shown to pave the way for the evolution of chromosomal mutations conferring antibiotic resistance [37]. Our data raises the reassuring possibility that the spread of such antibiotic resistant bacteria to other patients may be limited by the significant fitness cost of the tolerance mutations, at least in the absence of reversion to WT or the accumulation of additional compensatory mutations. It is noteworthy that these tolerant populations still could act as a reservoir for antimicrobial resistance since they could exchange plasmid-encoded resistance genes by horizontal gene transfer [9].

In conclusion, our data depict the trade-offs of two different types of recalcitrance (Fig 6). When it comes to persistence, the balance is shifted towards proliferation as most of the population can grow while a subpopulation of cells acts as a reservoir for recalcitrance, minimizing the fitness cost of this latter feature. In contrast, the equilibrium is shifted towards antibiotic survival in a tolerant population as cells are all kept in the same recalcitrant state. Even if antibiotic tolerance can transiently allow a population to escape the action of a bactericidal drug, persistence remains a more balanced and versatile strategy overall in the face of multiple stresses encountered by bacteria during infection. Ultimately, a better understanding of antibiotic persistence and tolerance as well as the identification of their respective determinants during infection will contribute to the development of more efficient antibiotic treatments to limit repeated treatment failure.

## Material and methods

### Bacterial strains and plasmids

Oligonucleotides, strains and plasmids used in this study are listed in Tables A, B and C, together with construction details provided in S1 Supplementary Methods. *Escherichia coli* DH5α and *Salmonella* strains used in this study were grown aerobically in Luria-Bertani (LB) broth (Invitrogen) or in synthetic M9 medium (1x M9 salts, Sigma; 2mM MgSO_4_, Sigma; 0.1mM CaCl_2_, Sigma) containing 0.1% casamino acids (BD), 0.2% glucose (Sigma), 0.2% glycerol (Sigma), 0.2% arabinose (MP Biomedical) and/or 1mM histidine (Sigma) when indicated. Antibiotics were used at the following concentrations: carbenicillin (100 μg/ml, CHEM-IMPEX INT’L INC), kanamycin (50 μg/ml, Sigma), and chloramphenicol (34 μg/ml, CHEM-IMPEX INT’L INC). For mutants generated by λ-Red recombination, the pKD4 plasmid was used as the template to amplify the kanamycin resistance cassette, and the amplification reaction products were transferred by electroporation into pKD46-containing bacteria expressing the λ-Red recombinase, as described previously [38]. Then, the antibiotic resistance cassette of interest was transduced onto WT *Salmonella enterica* serovar Typhimurium strain 12023/14028 using P22 bacteriophage and then confirmed by PCR. Finally, the kanamycin cassette was excised using the temperature-inducible FLP recombinase encoded on the pCP20 recombination plasmid [38].

### *In vitro* growth assay

Bacteria were grown overnight in minimal medium containing glucose and glycerol (M9GG) and supplemented with 1mM histidine—when indicated—to allow the growth of the auxotrophic strains. Bacterial cultures were washed twice with M9GG to remove residual histidine. Then, serial dilutions (10x) made in M9GG were plated onto M9GG solid media in presence or in absence of histidine and incubated at 37°C for 16 h.

### *Salmonella* antibiotic survival assays *in vitro*

Bacteria were grown overnight in M9GG supplemented with 1mM histidine. Stationary phase bacteria were diluted 1:10 into M9GG media with or without histidine and incubated in a 37°C shacking incubator (220 rpm). After 30 min, samples were collected and CFU enumerated (T = 0 h). Cefotaxime (10 μg/ml) was added to the medium and cultures were incubated at 37°C. 16 and 24 h after the addition of the antibiotic, samples were taken and CFU enumerated (T = 16 h and 24 h) (Fig 1B). For Fig 1E, bacteria were directly resuspended in M9 medium without carbon sources. For Fig 1F, chloramphenicol was added at the same time as cefotaxime.

### *Salmonella* fluorescence dilution assay *in vitro*

*Salmonella* strains containing the chromosomally-encoded fluorescence dilution reporter (S1A Fig) were grown overnight in M9 medium containing glycerol, arabinose, and histidine–when indicated—to induce the production of mCherry. Then, bacterial cultures were diluted 1:100 into M9GG and incubated in a 37°C shacking incubator. After 30 min, samples were taken (T = 0h) and stored at 4°C in PBS prior analysis. After 4 h, samples were taken (T = 4h) and analysed on a BD LSR II flow cytometer (Fig 2A). Constitutive GFP was used to discriminate bacteria in the samples from debris.

### Bone marrow-derived macrophages derivation and culture

Extraction and culture of bone marrow macrophages was performed as previously described [39]. Bone marrow was extracted from tibias and femurs of C57BL/6 female mice (Jackson Lab) older than 8 weeks. After isolating both bones from both legs, bone marrow was flushed out of each cut bone using 23Gx3/4 needle (BD). Red blood cells were lysed in 0.83% freshly prepared NH_4_Cl (Sigma) for 3 min and the remaining cells were seeded in 100 mm non-tissue culture treated plates (Corning) at a concentration of 3E+6 cells per plates in 8 ml of Dulbecco’s modified eagle medium with high glucose (DMEM; Corning) containing 20% (vol/vol) of L929 supernatant (LCM), 10% (vol/vol) of fetal bovine serum (FBS; Premium Select from R&D Systems), 10mM of HEPES (Sigma), 1mM of sodium pyruvate (Sigma), 0.05mM of β-mercaptoethanol (Sigma) and 100 U/ml of penicillin/streptomycin (Genesee Scientific). After 3 days, 10 ml of fresh medium was supplemented and the differentiated bone marrow derived macrophages were harvested 4 days later, on day 7. Macrophages were seeded in DMEM media supplemented with FBS, HEPES, sodium pyruvate and β-mercaptoethanol but without LCM or antibiotics in 6-well tissue culture treated plates at the concentration of 1E+6 macrophages per well if freshly harvested or 1.2E+6 macrophages per well if from frozen stock, to be infected the next day.

### Macrophages infections and bacterial extraction

Macrophages infections and bacterial extraction were performed as previously described [40]. Bacteria were grown in LB medium (Invitrogen) for 16 h. Stationary phase bacteria were then opsonized with mouse serum (Sigma) and added to the macrophages at a Multiplicity of Infection (MOI) of 10. Synchronization of the infection was performed by centrifugation (5 min; 110 x *g*). Infected macrophages were then incubated for 30 min at 37°C with 5% CO_2_ to allow bacterial internalization. Macrophage media was exchanged with fresh media containing either cefotaxime (100 μg/ml; TCI) or gentamycin (50 μg/ml for the first 30 min then replaced and kept at 10 μg/ml; Sigma) to test intramacrophage bacterial antibiotic survival or bacterial proliferation, respectively. At selected time points, infected macrophages were washed three times with PBS (Growcells) and lysed using 0.1% Triton X-100 (Sigma) to extract intracellular bacteria. Bacteria were collected, centrifuged for 3 min at 16,000 x *g* and resuspend in PBS prior further experiments.

### Fluorescence dilution analysis of intramacrophagic *Salmonella*

Fluorescence dilution experiments were performed as previously described [40]. Briefly, *Salmonella* strains containing the chromosomally-encoded fluorescence dilution reporter (S1A Fig) were grown overnight in M9 medium containing glycerol, arabinose and histidine to induce the production of mCherry. Bacteria were used to infect macrophages (as described above) in the absence of cefotaxime to allow bacterial proliferation (S2B Fig). At 16 h post-infection, bacteria were extracted from macrophages and analysed on a BD LSR II flow cytometer. Constitutive GFP was used to discriminate bacteria in the samples from debris. For fluorescence dilution experiments in the SL1344 strain (S1C and S1E Fig), bacteria containing the pFCcGi reporter were used as previously described [40].

### *Salmonella* antibiotic survival assays in infected macrophages

Antibiotic survival assays were performed as previously described [40]. To assess the number of bacteria within macrophages after 30 min of invasion and before antibiotic treatment, part of the infected macrophages was washed three times with 1x PBS, lysed with 0.1% Triton X-100 (Sigma) in PBS. Lysed infected macrophages were collected, centrifuged for 3 min at 16,000 x *g* at room temperature and resuspended in PBS. Ten-fold serial dilutions in PBS were performed and drops of 20 μl were plated on LB agar to determine the number of CFU at T0. Antibiotic treatment was applied to the rest of the infected macrophages. Macrophages media was exchanged with fresh media containing either cefotaxime (100 μg/ml; TCI) or gentamycin (50 μg/ml for the first 30 min then replaced and kept at 10 μg/ml; Sigma) to test intramacrophage bacterial antibiotic survival or bacterial proliferation, respectively. Histidine at the concentration of 2mM was added to the cefotaxime media when indicated (Histidine HCl, Sigma). For cefotaxime, after 2 h, 24 h and 48 h of antibiotic treatment, infected macrophages were washed three times with 1xPBS and lysed using 0.1% Triton X-100. Bacteria were collected, centrifuged for 3 min at 16,000 x *g*, resuspended in PBS and either ten-fold serial diluted for the 2 h time point or plated entirely in LB agar plate to assess antibiotic survival. For gentamycin, after 24 h of antibiotic treatment, infected macrophages were washed three times with PBS and lysed using 0.1% Triton X-100. Bacteria were collected, centrifuged for 3 min at 16,000 x *g*, resuspended in PBS and ten-fold serial diluted. 20 μl drops were plated into LB agar to assess bacterial proliferation. For complementation of the *shpAB1* strain (S3A Fig), expression of *shpB* relied on the basal activity of the P*lac* promoter of the pCA24N plasmid.

### Competitive assays in mice

The three strains used—GFP+ (WT) and mCherry+ bacteria (*shpAB1* or *ΔhisG*)—were grown overnight separately for 16 h without antibiotics in LB (Invitrogen) prior to infection. 8 to 10 week-old SPF C57BL/6 mice (Jackson Laboratories) were housed in groups of 5 and were inoculated by gavage with 200 μl of PBS containing approximately 1E+10 CFU of combined WT and *ΔhisG* or WT and *shpAB1* at a ratio of 1:1. Three cages of 5 animals each received each mixture. One cage per mix was sacrificed 48 h post-infection. Mice were culled by carbon monoxide asphyxiation followed by cervical dislocation. The other cages received cefotaxime treatment (150 mg/kg, administered intraperitoneally every 12 h) for 4 or 6 days. After completion of the 4 or 6 days of treatment, one cage per time point and per mix was sacrificed. For each time point, the spleens, mesenteric lymph nodes and 2 Payer patches per animal were collected and mechanically homogenised in cold PBS using screw cap 1.5 ml tubes (USA Scientific) containing three 3.2 mm steel beads (Biospec). Organs were homogenized (Retsch MM400) for 3 min at 30 Hz. Afterwards, homogenates were centrifuged for 3 min at 16000 x *g*, and the pellets were resuspended in cold water to lyse the mammalian cells. For the acute phase, the samples were diluted in PBS and dilutions were plated into LB agar (20 μl drops). For the 4 and 6 days antibiotic treatment time points, the whole resuspended organs were plated into LB agar. Plates were all incubated at 37°C to count the number of CFU in each organ. GFP and mCherry colonies were visualized using a ChemiDoc Imaging System (BioRad) and enumerated using the ImageJ software. Mice were housed with sterile bedding and nesting and received autoclaved chow and water over the course of the study. All experiments involving mice were pre-reviewed and approved by the Harvard Medical School Institutional Animal Care and Use Committee (IACUC).

## Supporting information

S1 Fig Fluorescence dilution facilitates the distinction between antibiotic tolerance and persistence. (**A**) Illustration of the fluorescence dilution principle. Both *mcherry* and *sfgfp* are encoded on the *Salmonella* genome. mCherry expression is driven by an arabinose-inducible promoter and GFP by a constitutive promoter. When expression of mCherry is induced by arabinose, the whole population is both green and red. After removal of the inducer, growing bacteria dilute the mCherry pool at each division whereas non-growers retain red and green fluorescence. (**B**) Illustration of fluorescence dilution within macrophages. (**C**) Representative flow cytometry contour (left) and histogram plots (right) of the SL1344 strain extracted from infected BMDM after 16 h of gentamicin in presence or absence of histidine. G, growers and NG, non-growers. (**D**) Quantification of the non-grower fraction in presence or in absence of histidine. Data represent the mean and SD of three biological repeats. Statistical significant difference by *t*-test are indicated as ***p<0.001.
(PDF)

S2 Fig Growth of 14028 and SL1344 *Salmonella* strains on laboratory medium. Ten-fold dilution series were spotted on minimal medium plates in the absence (left) or presence (right) of histidine for (**A**) WT, *ΔhisG*, *shpAB1* and *shpAB1ΔhisG* or (**B**) WT, *ΔhisG*, *ΔrecA* and *ΔrecA ΔhisG*.
(PDF)

S3 Fig Additional *in cellulo* antibiotic survival profiles of the strains used in the study. (**A**) 24 h cefotaxime survival of WT and *shpAB1 Salmonella* in BMDM normalized to values after 30 min internalization. WT and *shpAB1* strains were complemented with an empty vector (pEV) or *shpB* (p*shpB*). Data represent the mean and SD of three biological repeats. Data were compared using one way ANOVA with Tukey’s multiple-comparison test, **p<0.01. (**B-C**) CFU enumeration of survival of WT, *ΔhisG*, *shpAB1* and *shpAB1ΔhisG* (**B**) or WT, *ΔhisG*, *ΔrecA* and *ΔrecA ΔhisG* (**C**) to cefotaxime during BMDM infection in the presence of histidine in the infection medium. Data represent the mean and SD of at least three biological repeats. Data from the 48 h timepoint were compared using one way ANOVA with Tukey’s multiple-comparison test, *p<0.05, **p<0.01 ***p<0.001.
(PDF)

S4 Fig *In vivo* competitive assays of WT/ *ΔhisG* and WT/*shpAB1* mixtures in MLN and Peyer’s patches. (**A-D**) Bacterial survival in the mesenteric lymph nodes and in the Peyer’s patches of the WT/*ΔhisG* (**A-B**) and WT/*shpAB1* (**C-D**) mix. After two days of infection in the absence of antibiotic (-AB), mice were treated for 4 to 6 days with cefotaxime (+AB). Results are expressed as percentage of the total population recovered on agar plates after 24 h incubation. Statistically significant differences at each timepoint by two-sided *t*-test are indicated as *p<0.05, * p<0.01, ***p<0.001; NS, not significant. *In vivo* mice experiments were carried out in at least 4 animals per time point.
(PDF)

S5 Fig Total amount of bacteria recovered from the spleen, the MLN and the Peyer’s patches after infection by WT/ *ΔhisG* and WT/*shpAB1* mixtures. **(A-B)** Bacterial survival in the spleen, the mesenteric lymph nodes and in the Peyer’s patches of the WT/*ΔhisG* (**A**) and WT/*shpAB1* (**B**) mix. After two days of infection in the absence of antibiotic, mice were treated for 4 to 6 days with cefotaxime. Results are expressed as the total number of bacteria per organ recovered on agar plates after 24 h incubation.
(PDF)

S1 Supplementary methods Can be found in supporting information. **Table A**. Oligonucleotides used in this study. **Table B**. Plasmids used in this study. **Table C**. Strains used in this study.
(DOCX)
