# Supplementary figures and images for: Antibiotic tolerance and persistence have distinct fitness trade-offs
Source: PLoS Pathog. 2022 Nov 14;18(11):e1010963. doi: 10.1371/journal.ppat.1010963 (PMC9704765; doi:10.1371/journal.ppat.1010963)

# S1 Fig

**A**

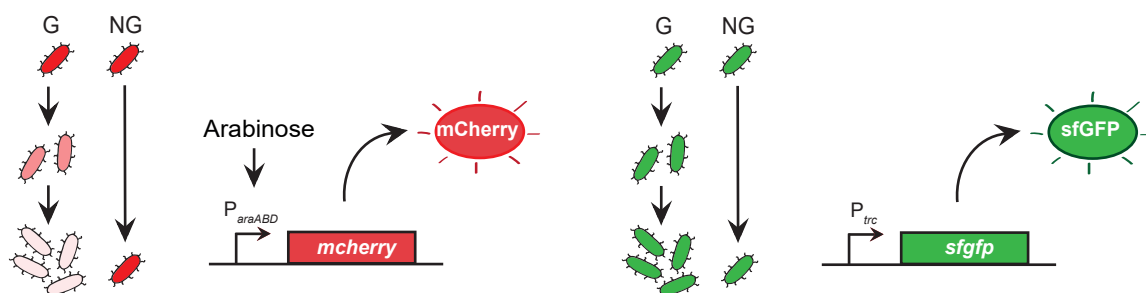

**B**

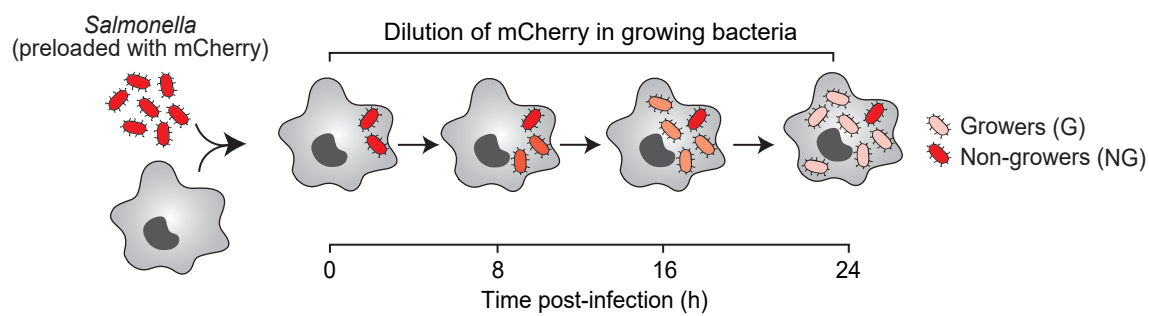

**C**

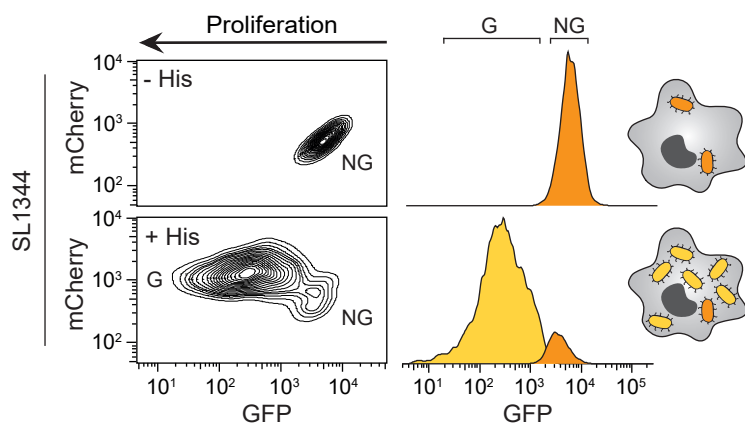

**D**

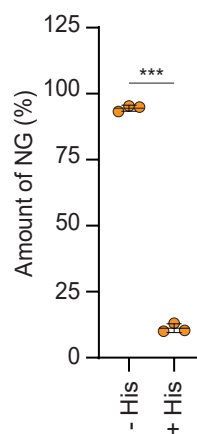

Supplement: S1 Fig — (A) Illustration of the fluorescence dilution principle. Both mcherry and sfgfp are encoded on the Salmonella genome. mCherry expression is driven by an arabinose-inducible promoter and GFP by a constitutive promoter. When expression of mCherry is induced by arabinose, the whole population is both green and red. After removal of the inducer, growing bacteria dilute the mCherry pool at each division whereas non-growers retain red and green fluorescence. (B) Illustration of fluorescence dilution within macrophages. (C) Representative flow cytometry contour (left) and histogram plots (right) of the SL1344 strain extracted from infected BMDM after 16 h of gentamicin in presence or absence of histidine. G, growers and NG, non-growers. (D) Quantification of the non-grower fraction in presence or in absence of histidine. Data represent the mean and SD of three biological repeats. Statistical significant difference by t-test are indicated as ***p<0.001. (PDF) [file ppat.1010963.s001.pdf]

# S2 Fig

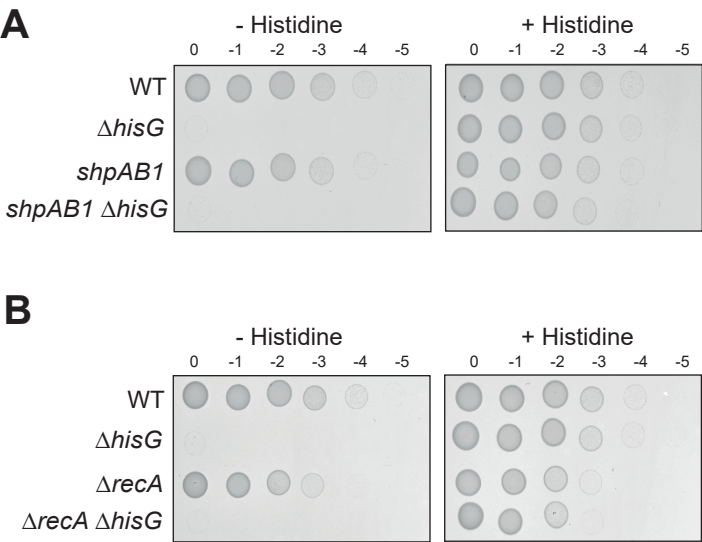

Supplement: S2 Fig — Ten-fold dilution series were spotted on minimal medium plates in the absence (left) or presence (right) of histidine for (A) WT, ΔhisG, shpAB1 and shpAB1ΔhisG or (B) WT, ΔhisG, ΔrecA and ΔrecA ΔhisG. (PDF) [file ppat.1010963.s002.pdf]

## S3 Fig

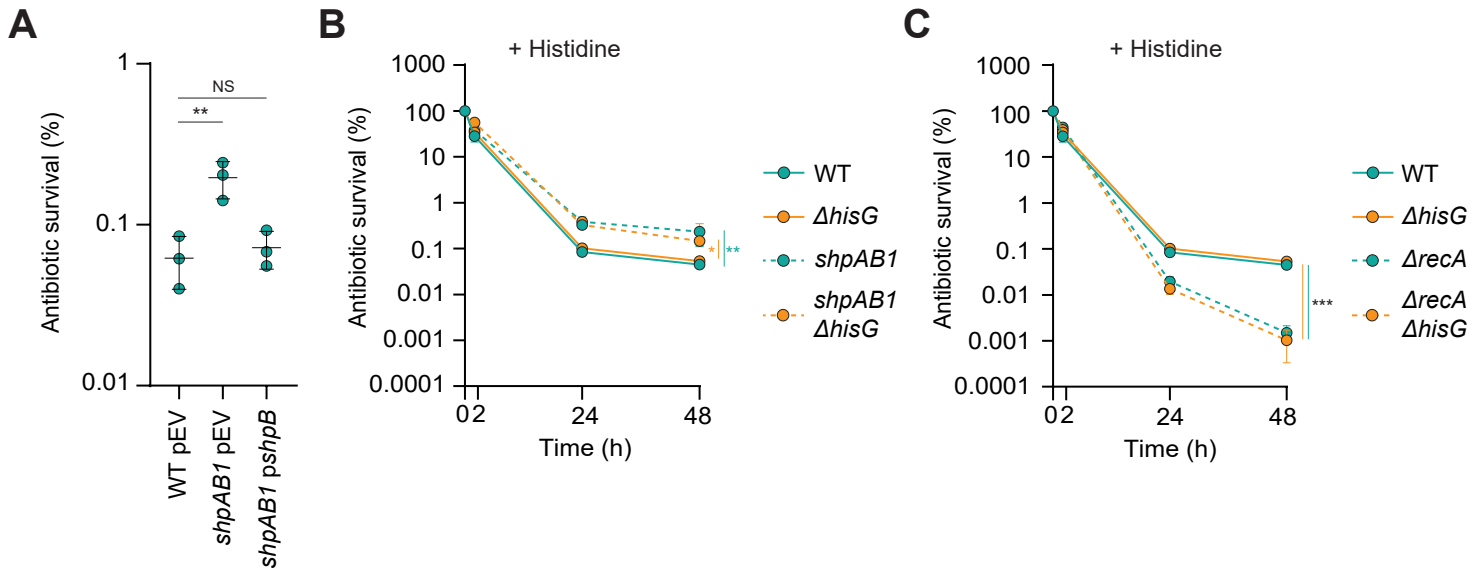

Supplement: S3 Fig — (A) 24 h cefotaxime survival of WT and shpAB1 Salmonella in BMDM normalized to values after 30 min internalization. WT and shpAB1 strains were complemented with an empty vector (pEV) or shpB (pshpB). Data represent the mean and SD of three biological repeats. Data were compared using one way ANOVA with Tukey’s multiple-comparison test, **p<0.01. (B-C) CFU enumeration of survival of WT, ΔhisG, shpAB1 and shpAB1ΔhisG (B) or WT, ΔhisG, ΔrecA and ΔrecA ΔhisG (C) to cefotaxime during BMDM infection in the presence of histidine in the infection medium. Data represent the mean and SD of at least three biological repeats. Data from the 48 h timepoint were compared using one way ANOVA with Tukey’s multiple-comparison test, *p<0.05, **p<0.01 ***p<0.001. (PDF) [file ppat.1010963.s003.pdf]

## S4 Fig

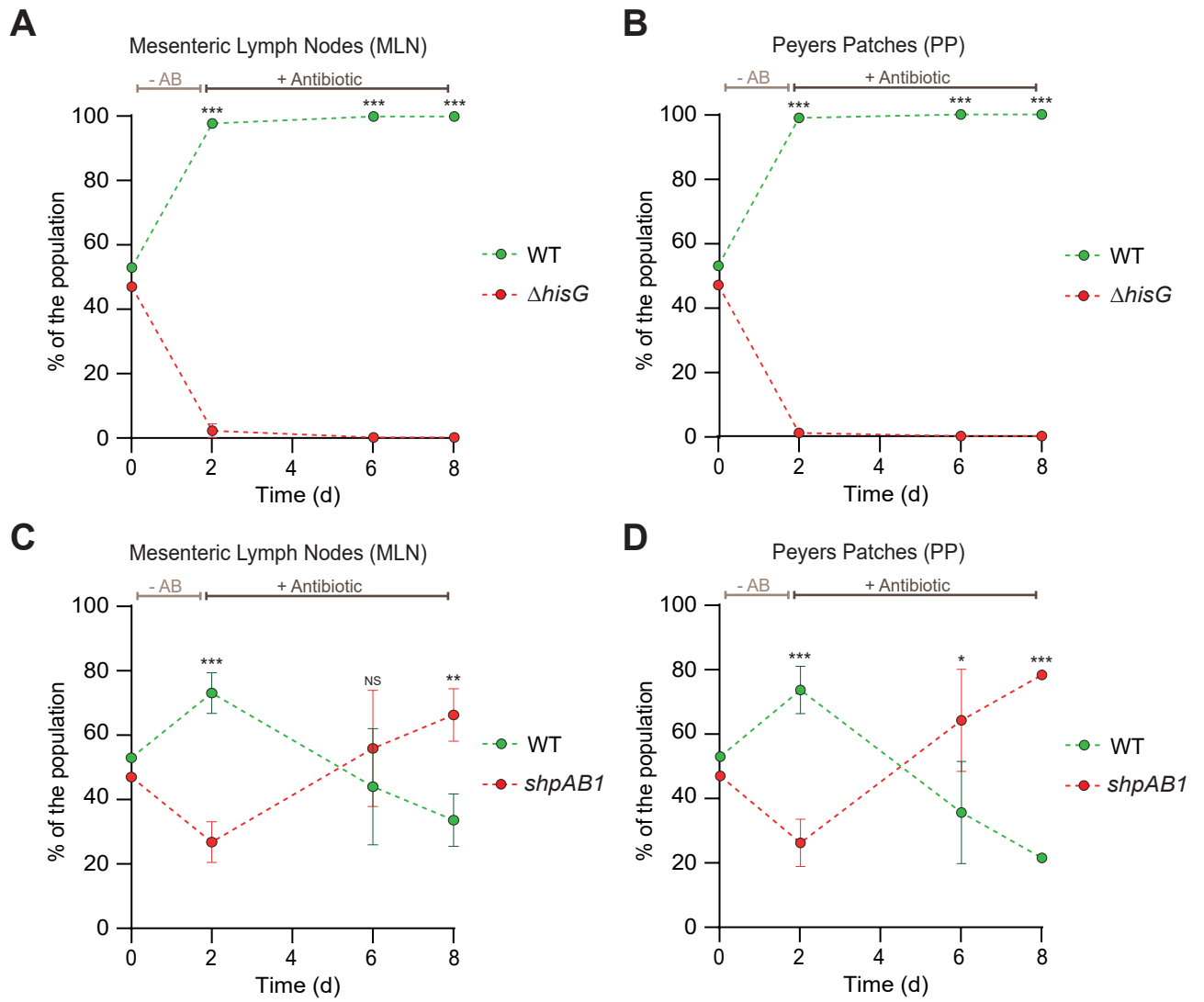

Supplement: S4 Fig — (A-D) Bacterial survival in the mesenteric lymph nodes and in the Peyer’s patches of the WT/ΔhisG (A-B) and WT/shpAB1 (C-D) mix. After two days of infection in the absence of antibiotic (-AB), mice were treated for 4 to 6 days with cefotaxime (+AB). Results are expressed as percentage of the total population recovered on agar plates after 24 h incubation. Statistically significant differences at each timepoint by two-sided t-test are indicated as *p<0.05, * p<0.01, ***p<0.001; NS, not significant. In vivo mice experiments were carried out in at least 4 animals per time point. (PDF) [file ppat.1010963.s004.pdf]

## S5 Fig

**A**

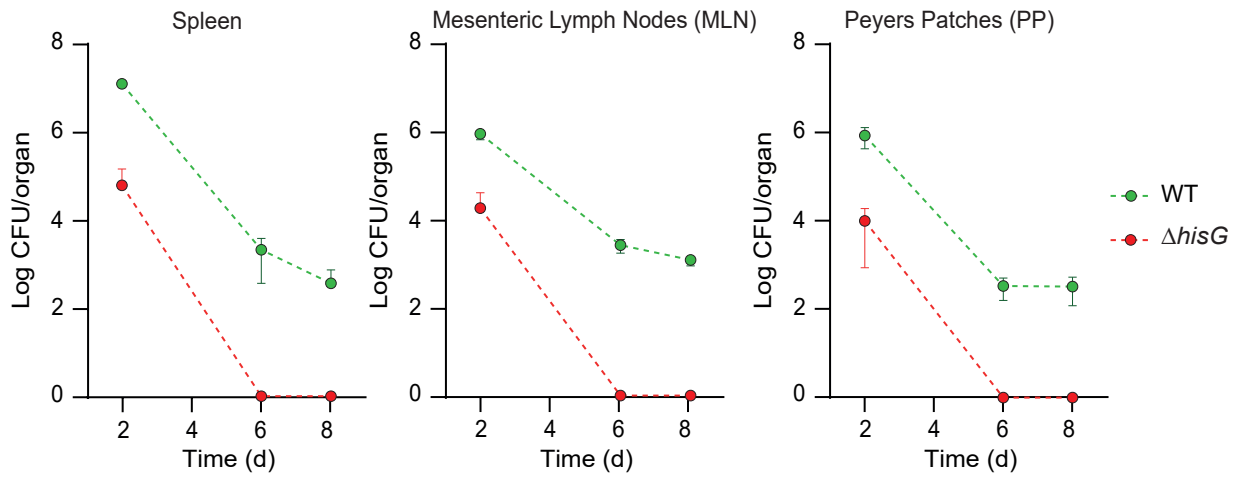

**B**

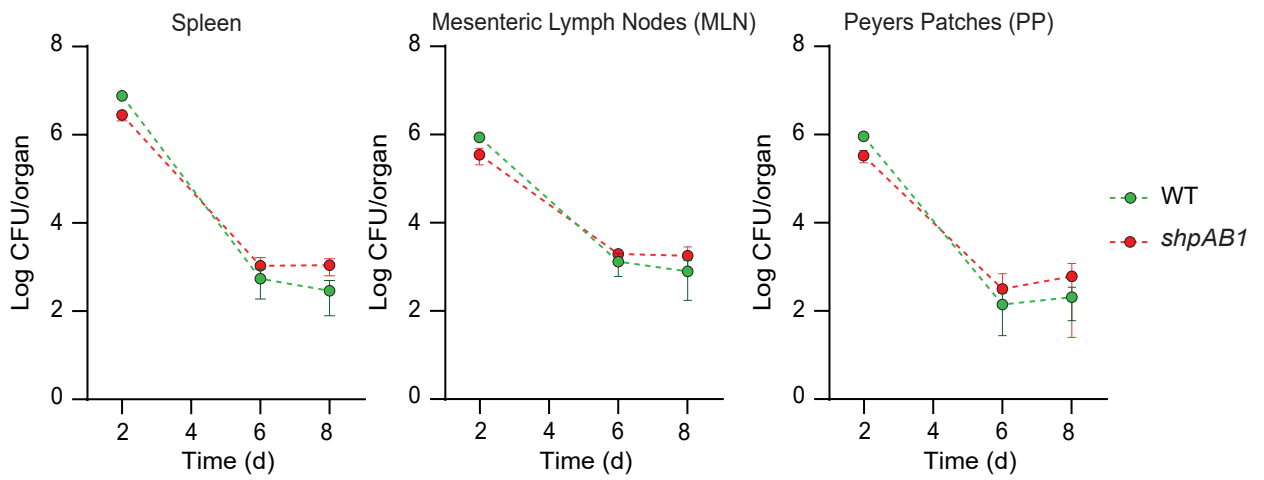

Supplement: S5 Fig — (A-B) Bacterial survival in the spleen, the mesenteric lymph nodes and in the Peyer’s patches of the WT/ΔhisG (A) and WT/shpAB1 (B) mix. After two days of infection in the absence of antibiotic, mice were treated for 4 to 6 days with cefotaxime. Results are expressed as the total number of bacteria per organ recovered on agar plates after 24 h incubation. (PDF) [file ppat.1010963.s005.pdf]
