# Supplementary material for: Antibiotic tolerance and persistence have distinct fitness trade-offs
Source: PLoS Pathog. 2022 Nov 14;18(11):e1010963. doi: 10.1371/journal.ppat.1010963 (PMC9704765; doi:10.1371/journal.ppat.1010963)
Supplement: S1 Supplementary methods — Table A. Oligonucleotides used in this study. Table B. Plasmids used in this study. Table C. Strains used in this study. (DOCX) [file ppat.1010963.s006.docx]

**Supplementary Tables**

**Table A.** Oligonucleotides used in this study.

| Name | Sequence |
| --- | --- |
| **oSR53** | GGATCCCCGGGTACAATTGATGAGCATGGTTAAACATAAACGCGGCAACGCATC |
| **oSR54** | CCTAAGTAACTAAAAGCTTTCCGGCATCTTCGGGCTATT |
| **oSR73** | ACTGTTTCTCCATACCTGTTTTTCTGGATGGAGTAAGACGATGGTTAGCAAAGGTGAAGAAGAC |
| **oSR74** | CCCGGTAAACGCAGTGCCACCGGGCAATCCGTTTCACCAAAAATCCGCCGCTAGGAGCTT |
| **oSR377** | CAGACGCGGCTCAATGCCATTCTGCGCGAGGCGATGCTGCGCGAGTAAAATAAGAAATAGGTGTAGGCTGGAGCTGCTTC |
| **oSR378** | TCTGGCCTGGCGGGAATTTGTAGGCCGGATAAGGCGCAGCCGCCATCCGGCATCTTCGGGCATATGAATATCCTCCTTA |

**Table B.** Plasmids used in this study.

| Name | Description | Reference |
| --- | --- | --- |
| **pRS22** | pKD4 | [1] |
| **pRS24** | pCP20 | [1] |
| **pRS33** | pKD46 | [1] |
| **pRS43** | pCA24N_shpB (p*shpB*) | This study |
| **pRS626** | pFCcGi | [2] |
| **pRS631** | pCA24N (pEV) | [3] |

**Table C.** Strains used in this study.

| Name | Description and relevant genotype | Source |
| --- | --- | --- |
| **RS1** | *Salmonella enterica* serovar Typhimurium strain 12023/14028 | ATCC – strain designation CDC 6516-60 |
| **RS76** | *Salmonella enterica* serovar Typhimurium strain 12023/14028 *recA::kan* | [4] |
| **RS88** | *Salmonella enterica* serovar Typhimurium strain 12023/14028 *glmS::sfgfp* | [5] |
| **RS89** | *Salmonella enterica* serovar Typhimurium strain 12023/14028 *glmS::mcherry* | [5] |
| **RS213** | *Salmonella enterica* serovar Typhimurium strain 12023/14028 *araAB::mcherry glmS::sfgfp* | This study |
| **RS269** | *Salmonella enterica* serovar Typhimurium strain 12023/14028 *shpAB1* | This study |
| **RS438** | *Salmonella enterica* serovar Typhimurium strain 12023/14028 *hisG::kan* | [6] |
| **RS491** | *Salmonella enterica* serovar Typhimurium strain SL1344 pFCcGi | This study |
| **RS497** | *Salmonella enterica* serovar Typhimurium strain 12023/14028 *araAB::mcherry glmS::sfgfp shpAB1* | This study |
| **RS514** | *Salmonella enterica* serovar Typhimurium strain 12023/14028 *glmS::mcherry shpAB1* pCA24N | This study |
| **RS515** | *Salmonella enterica* serovar Typhimurium strain 12023/14028 *glmS::mcherry shpAB1* pCA24N_*shpB* | This study |
| **RS564** | *Salmonella enterica* serovar Typhimurium strain 12023/14028 *glmS::mcherry shpAB1* | This study |
| **RS631** | *Salmonella enterica* serovar Typhimurium strain 12023/14028 pCA24N | This study |
| **RS858** | *Salmonella enterica* serovar Typhimurium strain 12023/14028 *shpAB1 hisG::kan* | This study |
| **RS1259** | *Salmonella enterica* serovar Typhimurium strain 12023/14028 *araAB::mcherry glmS::sfgfp hisG::kan* | This study |
| **RS1260** | *Salmonella enterica* serovar Typhimurium strain 12023/14028 *araAB::mcherry glmS::sfgfp shpAB1 hisG::kan* | This study |
| **RS1262** | *Salmonella enterica* serovar Typhimurium strain 12023/14028 Δ*hisG recA::kan* | This study |
| **RS1273** | *Salmonella enterica* serovar Typhimurium strain 12023/14028 *araAB::mcherry glmS::sfgfp recA::kan* | This study |
| **RS1274** | *Salmonella enterica* serovar Typhimurium strain 12023/14028 *araAB::mcherry glmS::sfgfp* Δ*hisG recA::kan* | This study |
| **RS1281** | *Salmonella enterica* serovar Typhimurium strain 12023/14028 *glmS::mcherry recA::kan* | This study |
| **RS1331** | *Salmonella enterica* serovar Typhimurium strain 12023/14028 *glmS::sfgfp shpAB1* Δ*hisG* | This study |
| **RS1337** | *Salmonella enterica* serovar Typhimurium strain 12023/14028 *glmS::mcherry* Δ*hisG* | This study |
| **RS1338** | *Salmonella enterica* serovar Typhimurium strain SL1344 | [7] |
| **RS1339** | *Salmonella enterica* serovar Typhimurium strain SL1344 *hisG^P69L^* | [7] |
| **RS1340** | *Salmonella enterica* serovar Typhimurium strain 12023/14028 *glmS::mcherry* Δ*hisG recA::kan* | This study |

**Supplementary Methods**

**Description of Plasmid**

**pRS43** (pCA24N_*shpB*)

The wild-type *Salmonella enterica* serovar Typhimurium strain 12023/14028 was used as a template to amplify the *shpB* gene by PCR with primers oSR53 and oSR54. The amplification product was transferred into the pCA24N using MfeI and HindIII restriction sites.

**Description of Strains**

**RS213** (12023/14028 *araAB::mcherry glmS::sfgfp*)

The strain 12023/14028 *glmS::mcherry_cat* (Knodler et al., 2014) was used as the template to amplify the *mcherry_cat* cassette by PCR with primers oSR73 and oSR74. The amplification reaction product was transferred into pKD46-containing bacteria expressing the λ-Red recombinase-expressing WT *Salmonella* (12023/14028). Then, the *mcherry_cat* cassette was transduced into RS88 (*Salmonella enterica* serovar Typhimurium strain 12023/14028 *glmS::sfgfp*) using P22 bacteriophage (Maloy, 1990). Finally, the chloramphenicol cassette was excised using the temperature inducible flipase recombinase (FLP) encoded on the pCP20 recombination plasmid [1].

**RS269** (12023/14028 *shpAB1*)

The pKD4 was used as the template to amplify the kanamycin cassette by PCR with primers oSR377 and oSR378 (containing the *shpB1* mutation). The amplification reaction product was transferred into pKD46-containing bacteria expressing the λ-Red recombinase-expressing WT *Salmonella* (12023/14028). Then, the cassette was transduced into WT *Salmonella* (12023/14028) using P22 bacteriophage (Maloy, 1990). Finally, the kanamycin cassette was excised using the temperature inducible FLP recombinase encoded on the pCP20 recombination plasmid [1].

**RS438** (12023/14028 *hisG::kan*)

The *hisG::kan* resistance cassette from the Single-Gene Deletion (SGD) collection [6] was transduced into RS1 (12023/14028) using P22 bacteriophage [8].

**RS497** (12023/14028 *araAB::mcherry glmS::sfgfp shpAB1*)

The *shpAB1::kan* resistance cassette from the SGD collection [6] was transduced into RS213 (12023/14028 *araAB::mcherry glmS::sfgfp*) using P22 bacteriophage [8]. Finally, the kanamycin cassette was excised using the temperature inducible FLP recombinase encoded on the pCP20 recombination plasmid [1].

**RS564** (12023/14028 *glmS::mcherry shpAB1*)

The *shpAB1::kan* resistance cassette was transduced into RS89 (12023/14028 *glmS::mcherry*) using P22 bacteriophage [8]. Finally, the kanamycin cassette was excised using the temperature inducible FLP recombinase encoded on the pCP20 recombination plasmid [1].

**RS858** (12023/14028 *shpAB1 hisG::kan*)

The *hisG::kan* resistance cassette from the SGD collection [6] was transduced into RS269 (12023/14028 *shpAB1*) using P22 bacteriophage [8].

**RS1259** (12023/14028 *araAB::mcherry glmS::sfgfp hisG::kan*)

The *hisG::kan* resistance cassette from the SGD collection [6] was transduced into RS213 (12023/14028 *araAB::mcherry glmS::sfgfp*) using P22 bacteriophage [8].

**RS1260** (12023/14028 *araAB::mcherry glmS::sfgfp shpAB1 hisG::kan*)

The *hisG::kan* resistance cassette from the SGD collection [6] was transduced into RS497 (12023/14028 *araAB::mcherry glmS::sfgfp shpAB1*) using P22 bacteriophage ([8].

**RS1262** (12023/14028 Δ*hisG recA::kan*)

The *recA::kan* resistance cassette from the SGD collection [6] was transduced into the pCP20-treated RS438 strain (12023/14028 Δ*hisG* strain) using P22 bacteriophage [8].

**RS1273** (12023/14028 *araAB::mcherry glmS::sfgfp recA::kan*)

The *recA::kan* resistance cassette from the SGD collection [6] was transduced into RS213 (12023/14028 *araAB::mcherry glmS::sfgfp*) using P22 bacteriophage [8].

**RS1274** (12023/14028 *araAB::mcherry glmS::sfgfp* Δ*hisG recA::kan*)

The *recA::kan* resistance cassette from the SGD collection [6] was transduced into the pCP20-treated RS1259 strain (12023/14028 *araAB::mcherry glmS::sfgfp* Δ*hisG)* using P22 bacteriophage [8].

**RS1281** (12023/14028 *glmS::mcherry recA::kan*)

The *recA::kan* resistance cassette from the SGD collection [6] was transduced into RS89 (12023/14028 *glmS::mcherry)* using P22 bacteriophage [8].

**RS1331** (12023/14028 *glmS::sfgfp shpAB1* Δ*hisG*)

The *glmS::sfgfp_cat* resistance cassette from the SGD collection [6] was transduced into the pCP20-treated RS858 strain (12023/14028 *shpAB1* Δ*hisG*) using P22 bacteriophage [8]. Finally, the kanamycin cassette was excised using the temperature inducible FLP recombinase encoded on the pCP20 recombination plasmid [6].

**RS1337** (*12023/14028 glmS::mcherry ΔhisG*)

The *hisG::kan* resistance cassette from the SGD collection [6] was transduced into the RS89 (12023/14028 *glmS::mcherry)* using P22 bacteriophage [8]. Finally, the kanamycin cassette was excised using the temperature inducible FLP recombinase encoded on the pCP20 recombination plasmid [1].

**RS1340** (12023/14028 *glmS::mcherry* Δ*hisG recA::kan*)

The *recA::kan* resistance cassette from the SGD collection [1] was transduced into RS1340 (12023/14028 *glmS::mcherry* Δ*hisG)* using P22 bacteriophage [8].

**References**

1. Datsenko, K.A., Wanner, B.L., 2000. One-step inactivation of chromosomal genes in *Escherichia coli* K-12 using PCR products. Proc Natl Acad Sci U S A 97, 6640–6645. https://doi.org/10.1073/pnas.120163297
2. Figueira, R., Watson, K.G., Holden, D.W., Helaine, S., 2013. Identification of *Salmonella* Pathogenicity Island Type III Secretion System Effectors Involved in Intramacrophage Replication of S. enterica Serovar Typhimurium: Implications for Rational Vaccine Design. mBio 4, e00065-13. https://doi.org/10.1128/mBio.00065-13
3. Kitagawa, M., Ara, T., Arifuzzaman, M., Ioka-Nakamichi, T., Inamoto, E., Toyonaga, H., Mori,

H., 2005. Complete set of ORF clones of *Escherichia coli* ASKA library (a complete set of E. coli K-12 ORF archive): unique resources for biological research. DNA Res 12, 291–299. https://doi.org/10.1093/dnares/dsi012

4. Hill, P.W.S., Moldoveanu, A.L., Sargen, M., Ronneau, S., Glegola-Madejska, I., Beetham, C., Fisher, R.A., Helaine, S., 2021. The vulnerable versatility of *Salmonella* antibiotic persisters during infection. Cell Host Microbe 29, 1757-1773.e10. https://doi.org/10.1016/j.chom.2021.10.002

5. Knodler, L.A., Crowley, S.M., Sham, H.P., Yang, H., Wrande, M., Ma, C., Ernst, R.K., Steele-Mortimer, O., Celli, J., Vallance, B.A., 2014. Noncanonical inflammasome activation of caspase-4/caspase-11 mediates epithelial defenses against enteric bacterial pathogens. Cell Host Microbe 16, 249–256. https://doi.org/10.1016/j.chom.2014.07.002

6. Porwollik, S., Santiviago, C.A., Cheng, P., Long, F., Desai, P., Fredlund, J., Srikumar, S., Silva, C.A., Chu, W., Chen, X., Canals, R., Reynolds, M.M., Bogomolnaya, L., Shields, C., Cui, P., Guo, J., Zheng, Y., Endicott-Yazdani, T., Yang, H.-J., Maple, A., Ragoza, Y., Blondel, C.J., Valenzuela, C., Andrews-Polymenis, H., McClelland, M., 2014. Defined single-gene and multi-gene deletion mutant collections in *Salmonella enterica* sv Typhimurium. PLoS One 9, e99820. https://doi.org/10.1371/journal.pone.0099820

7. Claudi, B., Spröte, P., Chirkova, A., Personnic, N., Zankl, J., Schürmann, N., Schmidt, A., Bumann, D., 2014. Phenotypic variation of *Salmonella* in host tissues delays eradication by antimicrobial chemotherapy. Cell 158, 722–733. https://doi.org/10.1016/j.cell.2014.06.045

8. Maloy, S.R., 1990. Experimental Techniques in Bacterial Genetics. Jones & Bartlett Learning.
